# Case Report: Extracorporeal membrane oxygenation in acute coronary syndrome: a rare case of massive left ventricular thrombus

**DOI:** 10.3389/fcvm.2026.1778609

**Published:** 2026-03-16

**Authors:** Jian Lan, Yifeng Mao, Chenyang Shi, Jian Ye, Qiang Zhong, Jingjing Li, Xijiang Zhang, Cheng Zheng

**Affiliations:** 1 Department of Critical Care Medicine, Taizhou Municipal Hospital (Affiliated Taizhou University Hospital), School of Medicine, Taizhou University, Taizhou, Zhejiang, China; 2 Department of Emergency Medicine, Taizhou Municipal Hospital (Affiliated Taizhou University Hospital), School of Medicine, Taizhou University, Taizhou, Zhejiang, China; 3 Department of Urology, Taizhou Central Hospital (Affiliated Hospital of Taizhou University), Taizhou, Zhejiang, China; 4 Taizhou Key Laboratory of Sepsis, Taizhou Municipal Hospital, Taizhou, China; 5 Taizhou Key Laboratory of Infection and Tumor Immunology, Taizhou Municipal Hospital, Taizhou, China

**Keywords:** anticoagulation, cardiogenic shock, left ventricular (LV) thrombus, LV unloading, myocardial infarction, veno-arterial extracorporeal membrane oxygenation (VA-ECMO)

## Abstract

**Background:**

Left ventricular (LV) thrombus is a catastrophic complication during veno arterial-extracorporeal membrane oxygenation (VA-ECMO) support for cardiogenic shock, arising from a confluence of hemodynamic stasis, a prothrombotic state, and potential limitations of conventional anticoagulation monitoring.

**Case presentation:**

A 42-year-old man with acute inferior-wall ST-elevation myocardial infarction developed refractory cardiogenic shock during percutaneous coronary intervention, necessitating VA-ECMO initiation. Dual antiplatelet therapy and systemic heparinization (targeting an activated partial thromboplastin time of 50–80 s) were maintained.

**Results:**

Despite therapeutic anticoagulation, serial echocardiography documented the formation of a massive LV thrombus occupying >90% of the cavity within a 14 h interval on the fourth day of ECMO support. The patient subsequently deteriorated into refractory multi-organ failure, leading to withdrawal of care.

**Conclusion:**

This case underscores the rapidity and severity of LV thrombus formation in VA-ECMO patients with severe ventricular dysfunction. It highlights the critical need for proactive management, including multimodal anticoagulation monitoring and aggressive, individualized LV unloading strategies that may require escalation beyond intra-aortic balloon pump support to more direct decompression methods.

## 1 Introduction

Extracorporeal membrane oxygenation (ECMO) serves as a pivotal salvage therapy for patients with refractory cardiogenic shock and respiratory failure, offering temporary cardiopulmonary support (1, 2). Despite its life-saving potential, ECMO is fraught with complications, among which thrombotic and hemorrhagic events are the most frequent and challenging to manage, with reported thrombosis rates as high as 52% (3). Left ventricular (LV) thrombus formation during veno-arterial (V-A) ECMO represents one of the most devastating thrombotic complications, carrying a mortality rate that approaches 100% in published series (4). Reported in up to 5% of VA-ECMO cases, its fulminant presentation in the early phase of support is particularly ominous and may be under-recognized (5). This case report describes the rapid development of a massive, near-total LV thrombus in a patient on VA-ECMO for post-infarction cardiogenic shock, despite conventional anticoagulation. We analyze the multifactorial pathophysiology and discuss imperative refinements in monitoring and management strategies to avert this fatal complication.

## 2 Case presentation

### 2.1 Chief complaint

A 42-year-old man presented to the emergency department with sudden-onset, severe substernal chest pain lasting 40 min.

### 2.2 History of present illness

**Figure 1:**
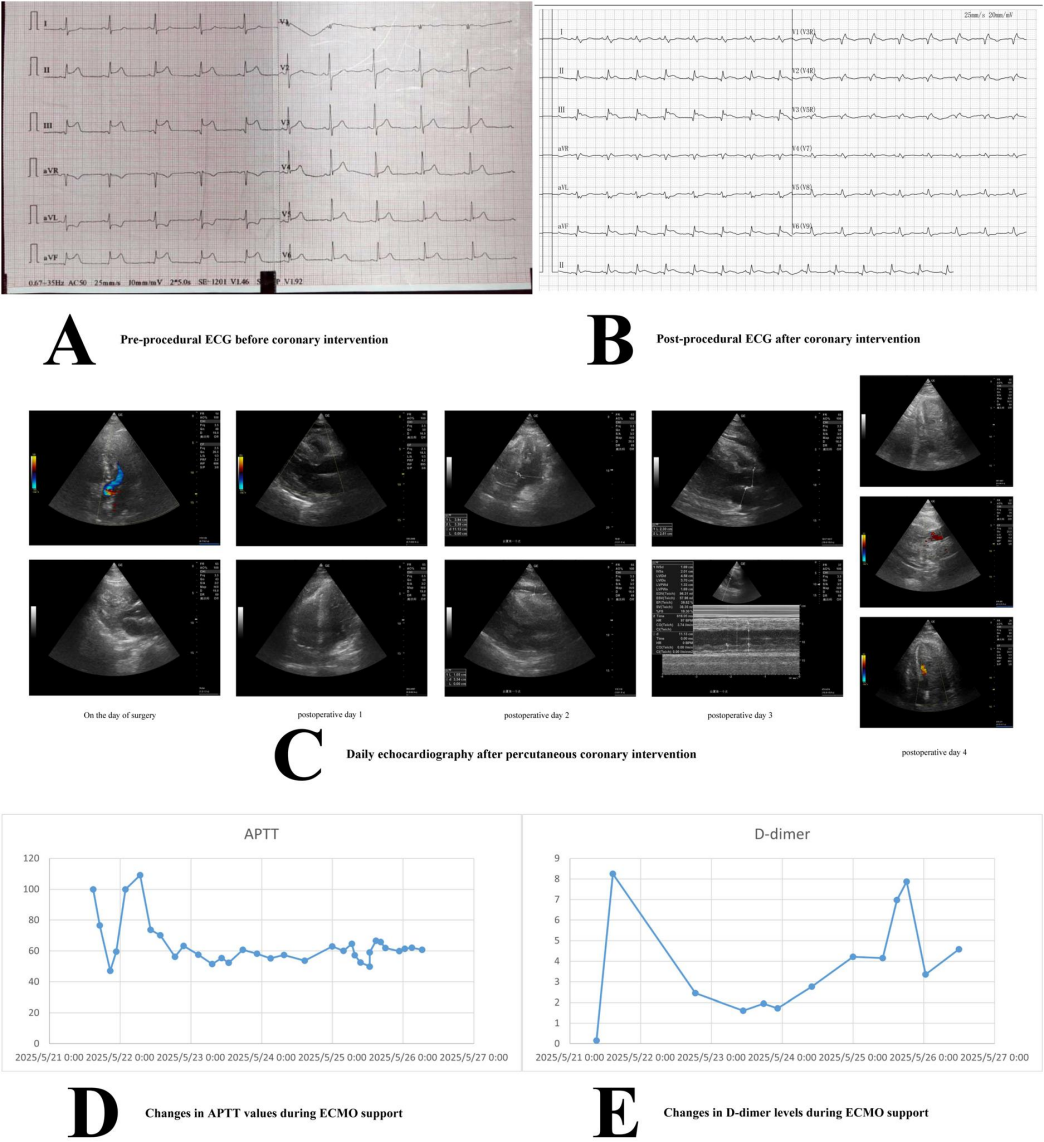
Imaging information during the treatment course. **(A)** Pre-procedural electrocardiogram showing ST-segment abnormalities. **(B)** Post-procedural electrocardiogram with normalized ST segments. **(C)** Daily echocardiogram images after percutaneous coronary intervention, illustrating cardiac function changes (color Doppler and M-mode included). **(D)** Activated partial thromboplastin time (APTT) values during extracorporeal membrane oxygenation (ECMO) support. **(E)** D-dimer levels during ECMO support.

The patient was in his usual state of health until the onset of pain. Upon arrival, his blood pressure was 127/89 mmHg. An initial 12-lead electrocardiogram (ECG) (Figure 1A) demonstrated ST-segment elevations in leads II, III, and aVF, consistent with an acute inferior-wall ST-elevation myocardial infarction (STEMI). Point-of-care laboratory testing revealed a markedly elevated high-sensitivity troponin T level of 9.992 µg/L. Serum creatinine was 86 µmol/L. After immediate loading with aspirin (300 mg) and ticagrelor (180 mg), the patient was transferred for emergency coronary angiography.

Coronary angiography revealed multi-vessel disease. The most critical lesion was a 99% stenosis in the proximal right coronary artery (RCA), which was deemed the culprit vessel. During percutaneous coronary intervention (PCI) of the RCA, the patient abruptly developed ventricular fibrillation. Prolonged advanced cardiac life support (ACLS), including endotracheal intubation, repeated defibrillation, and vasopressor infusion, was initiated. A cardiac arrest period of approximately 15–20 min preceded the establishment of adequate circulation. Due to persistent, refractory cardiogenic shock despite maximal medical therapy and return of spontaneous circulation, VA-ECMO was emergently instituted. The ECMO circuit was configured with a 21-French drainage cannula in the left femoral vein and a 17-French return cannula in the right femoral artery. An initial heparin bolus was administered, followed by a continuous infusion titrated to an activated partial thromboplastin time (APTT) target. Successful stent placement in the RCA was completed under ECMO support. The patient was then transferred to the intensive care unit (ICU) for ongoing management.

### 2.3 Past medical history

The patient had a 5-year history of hypertension, managed with regular antihypertensive medication (amlodipine 5 mg daily). There was no known history of diabetes, hyperlipidemia, prior cardiac events, or thromboembolic disease.

### 2.4 Physical examination on ICU admission

The patient was sedated, intubated, and mechanically ventilated. Vital signs under ECMO support showed a heart rate of 113 beats/min (predominantly ECMO-driven), and a respiratory rate set at 17 breaths/min. His core temperature was 36.1°C. Neurological examination was limited by sedation; pupils were equal and round (2 mm) with a sluggish light reflex. Cardiovascular examination revealed a quiet precordium with no palpable heaves. Heart sounds were distant; no murmurs, rubs, or gallops were audible. Bilateral coarse breath sounds were noted on lung auscultation. The abdomen was soft and non-tender. There was no peripheral edema.

### 2.5 Laboratory studies

Admission laboratory values were notable for a white blood cell count of 9.90 × 10^9^/L, platelet count of 283 × 10^9^/L, and C-reactive protein of 1.50 mg/L. Coagulation profile showed an APTT of 22.6 s and a D-dimer of 0.15 mg/L. Arterial blood gas on ICU admission revealed severe metabolic acidosis: pH 7.32, PaCO_2_ 26 mmHg, PaO_2_ 145 mmHg, base excess −11.1 mmol/L, lactate 13.6 mmol/L.

### 2.6 Imaging studies

The admission ECG was diagnostic for acute inferior ST-elevation myocardial infarction (Figure 1A). A follow-up ECG obtained post-intervention upon ICU admission showed improvement (Figure 1B). A concurrent bedside transthoracic echocardiogram revealed severe global left ventricular systolic dysfunction with marked chamber dilation and a severely reduced left ventricular outflow tract velocity-time integral of 9 cm/s. No intracardiac mass or thrombus was identified at this initial assessment.

### 2.7 Hospital course and management

Post-procedure management included continuation of dual antiplatelet therapy (aspirin and ticagrelor) and titration of the heparin infusion to maintain an APTT between 50 and 80 s. Two hours after ICU admission, the patient became anuric. Acute kidney injury progressed rapidly (creatinine rising to 143 µmol/L), necessitating the initiation of continuous renal replacement therapy (CRRT) connected in parallel to the ECMO circuit.

Four hours after ECMO initiation, the patient continued to exhibit a markedly low pulse pressure (<10 mmHg). Repeat transthoracic echocardiography (TTE) revealed persistent left ventricular distension, poor ejection, and an absence of aortic valve opening. In response, the ECMO pump speed was decreased to reduce left ventricular afterload, and an intra-aortic balloon pump (IABP) was inserted via the left femoral artery and set to 1:1 counter-pulsation to promote forward flow. These interventions led to a modest reduction in vasopressor requirements.

Approximately five hours after the PCI procedure, the ECMO flow began to decline despite stable circuit pressures. A systematic evaluation was undertaken: physical examination ruled out cannula dislodgement or kinking, clinical assessment and hemodynamic parameters suggested adequate volume status, and echocardiography excluded pericardial tamponade. With common causes of low flow addressed, inadequate venous drainage was suspected. To augment drainage, a second drainage cannula was placed in the right internal jugular vein, converting the circuit to a veno veno-arterial (VV-A) configuration, which successfully restored flows.

Over the next three days, the patient required ongoing support with blood products, inotropes, and antibiotics for suspected nosocomial pneumonia. Serial daily TTEs from post-operative days 1–3 showed some improvement in LV filling, with an estimated ejection fraction reaching 40% by day 3, and no evidence of intracardiac thrombus (Figure 1C). Vasopressor support was gradually weaned, and the intensity of IABP and ECMO support was cautiously reduced, though not discontinued.

On the morning of post-operative day 4, a routine bedside TTE was performed approximately 14 h prior to the subsequent catastrophic event. This examination revealed reduced left ventricular systolic function with an estimated ejection fraction of approximately 40%, increased left ventricular wall thickness, structurally normal cardiac valves with adequate opening and closure, normal atrial and ventricular chamber dimensions. Crucially, no intracardiac thrombus was identified at this time (Figure 1C).

Subsequently, the patient experienced an acute, catastrophic hemodynamic collapse. An emergent bedside TTE revealed a new, massive, laminated thrombus occupying over 90% of the LV cavity, with near-complete obliteration of the ventricular lumen (Figure 1C, Supplementary Video S1). The LV ejection fraction had plummeted to approximately 15%. Review of coagulation parameters showed the APTT had been consistently within the target range of 50–80 s (Figure 1D). However, D-dimer levels had shown a significant upward trend beginning 24 h prior to thrombus detection (Figure 1E).

**Figure 2:**
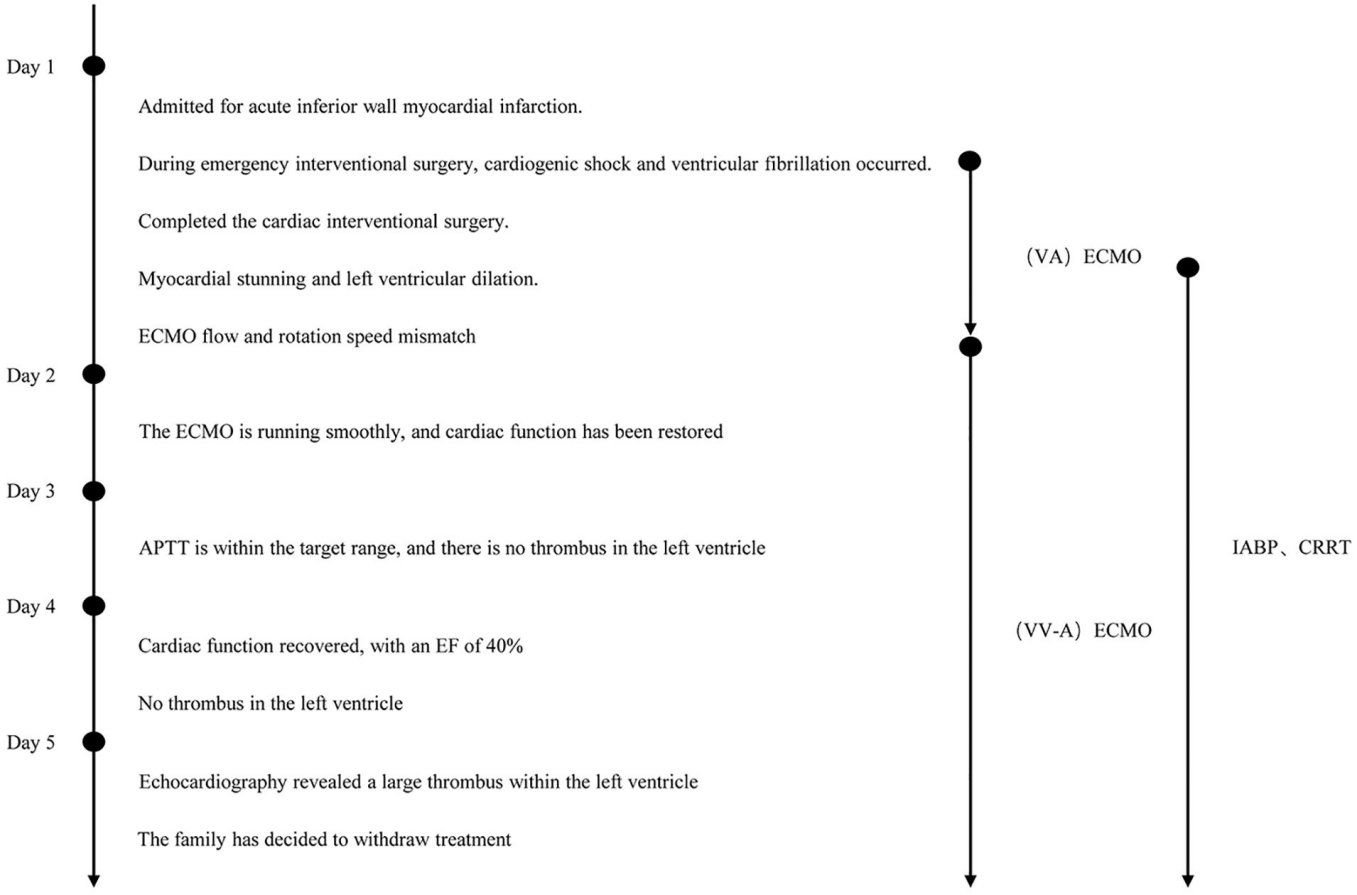
Timeline of the treatment course.

Faced with irreversible multi-organ failure (cardiac, renal, hepatic) and a universally poor prognosis, the family, after extensive counseling regarding the futility of further intervention and considering financial hardships, made the decision to withdraw life-sustaining therapies. The patient passed away shortly thereafter. A detailed timeline of the hospital course is presented in Figure 2.

### 2.8 Final diagnoses

Acute inferior-wall ST-elevation myocardial infarction, Refractory cardiogenic shock, Massive left ventricular thrombus, Multi-organ failure syndrome (cardiac, respiratory, renal, hepatic), Nosocomial pneumonia, Essential hypertension.

## 3 Discussion

This case presents a fulminant and fatal example of massive left ventricular thrombus complicating VA-ECMO support, a rare but devastating complication. The sheer rapidity of clot formation—developing to near-total cavity obliteration within 14 hours despite conventional anticoagulation—demands a critical re-examination of the converging pathophysiology and exposes significant limitations in current monitoring and management strategies.

An initial and crucial observation lies in the apparent discordance between the identified culprit lesion and the observed myocardial dysfunction. While angiography pinpointed a 99% proximal RCA stenosis, echocardiography revealed severe global LV systolic dysfunction with marked dilation, a pattern extending beyond the expected regional injury of an inferior MI. This suggests a multifactorial substrate. The patient's history of hypertension may have provided a background of adverse remodeling, reducing ventricular compliance and functional reserve (6, 7). The proximate 15–20 min cardiac arrest and resuscitation likely induced global myocardial stunning secondary to ischemic-reperfusion injury (8). Furthermore, in the setting of cardiogenic shock with low coronary perfusion pressure, even non-occlusive multi-vessel disease can contribute to a diffuse ischemic burden (9). Thus, the profound ventricular impairment was likely the product of a chronic substrate acutely destabilized by infarction, compounded by resuscitation-related injury and shock physiology. This created the foundational milieu—a severely dilated, hypocontractile ventricle—primed for the subsequent thrombotic catastrophe.

The thrombotic event can be pathophysiologically underpinned by Virchow's triad, encompassing endothelial injury from infarction, hemodynamic stasis within the dilated ventricle, and a clinically occult hypercoagulable state (10). The endothelial injury was initiated by the acute myocardial infarction. Stasis was dramatically potentiated by the hemodynamic effects of VA-ECMO (10). The retrograde arterial flow from the ECMO circuit increases LV afterload. In a ventricle already crippled by infarction—evidenced early on by severe dilatation and a VTI of only 9 cm/s—this elevated afterload can lead to profound distension and virtual cessation of ejection, creating a static blood pool (11–13). While our team appropriately responded to signs of LV distension by reducing ECMO flow and inserting an IABP, this case highlights the potential inadequacy of IABP as a sole unloading strategy in cases of severe ventricular dysfunction. IABP improves coronary perfusion and reduces aortic impedance but does not actively decompress the LV chamber or directly alleviate cavity stasis (14–16). For patients exhibiting such severe LV dilation and poor contractility on VA-ECMO, a more proactive approach to unloading may be warranted (17). This could involve the early use of or escalation to devices that provide direct LV decompression (18, 19).The Impella® microaxial pump, for instance, actively unloads the LV by pumping blood from the ventricle into the ascending aorta, effectively reducing ventricular volume and wall stress more definitively than IABP (20–22). Alternatively, percutaneous atrial septostomy can decompress the left heart by creating an interatrial shunt, though it may compromise oxygenation (23, 24). The decision must be individualized, but a low threshold for employing these more aggressive strategies should be considered when echocardiographic signs of persistent stasis (e.g., worsening dilation, spontaneous echo contrast) are present despite IABP support (17). In specialized centers, additional configurations such as left atrial veno-arterial (LAVA) ECMO or percutaneous pulmonary artery drainage may also be considered as alternative unloading strategies in select cases (23, 25, 26).

The third element, hypercoagulability, presents the most insidious challenge (10). Our patient maintained an APTT within the prescribed therapeutic range (50–80 s), yet developed a massive clot, illustrating the phenomenon of “heparin pseudo-adequacy”. (27, 28) This discrepancy can arise from several factors pervasive in ECMO patients. Acquired deficiency of antithrombin III (AT-III), a crucial cofactor for heparin, is common and leads to heparin resistance, rendering standard doses ineffective despite a normal APTT (29, 30). Furthermore, the APTT itself is a flawed monitor in this population; it is influenced by factor deficiencies, elevated factor VIII levels (common in critical illness), and the presence of lupus anticoagulants, all of which can distort its correlation with true heparin effect and thrombin generation (31–33). The rising D-dimer trend in our patient, though non-specific, was a biochemical red flag suggesting ongoing coagulation activation that APTT monitoring failed to capture (34, 35). This case strongly argues against sole reliance on APTT. Incorporating anti-factor Xa (anti-Xa) activity monitoring provides a more direct and reliable measure of heparin's anticoagulant effect and is less susceptible to the confounding variables that affect APTT (36). A multimodal monitoring protocol, incorporating APTT, targeted viscoelastic testing, and anti-Xa levels, alongside periodic assessment of AT-III activity in high-risk or non-responsive patients, represents a more comprehensive and rational approach to guiding anticoagulation therapy during ECMO.

This experience also informs a pragmatic approach to risk mitigation. While exhaustive thrombophilia screening (genetic and extensive phenotypic) is impractical in the emergent ECMO setting, a targeted strategy is feasible and necessary. Routine multimodal coagulation monitoring (APTT + anti-Xa) should be considered standard. Echocardiographic vigilance is paramount, with a low threshold for frequent exams in patients with dilated, poorly ejecting ventricles. LV unloading strategy must be dynamic; IABP is a reasonable first step, but its efficacy must be continuously assessed with clear criteria for escalation to more definitive unloading devices like Impella if stasis is not resolved (37).

In summary, massive LV thrombosis during VA-ECMO is not merely a rare misfortune but a potential culmination of identifiable and modifiable risk factors. This case serves as a critical reminder that conventional endpoints like APTT and supportive IABP may be insufficient guards against this complication in high-risk patients. A fundamental shifts towards early, aggressive LV unloading tailored to ventricular physiology, coupled with sophisticated, multimodal anticoagulation monitoring, is essential to improve outcomes for these critically ill patients.

## 4 Conclusion

We report a case of rapidly progressive, massive LV thrombus formation in a patient on VA-ECMO for post-STEMI cardiogenic shock, which proved fatal despite standard anticoagulation monitoring and IABP support. The case highlights the lethal synergy between VA-ECMO-induced ventricular stasis and an inadequately monitored prothrombotic state. It underscores the urgent need to move beyond traditional management silos by implementing proactive ventricular unloading strategies that address stasis directly and by adopting multimodal anticoagulation monitoring to ensure true therapeutic efficacy. Integrating these principles into standardized protocols for high-risk VA-ECMO patients could help prevent this devastating complication.

## Data Availability

The original contributions presented in the study are included in the article/Supplementary Material, further inquiries can be directed to the corresponding author.

## References

[1] Combes A Leprince P Luyt CE Bonnet N Trouillet JL Léger P Outcomes and long-term quality-of-life of patients supported by extracorporeal membrane oxygenation for refractory cardiogenic shock. Crit Care Med. (2008) 36(5):1404–11. 10.1097/CCM.0b013e31816f7cf7 18434909

[2] Smedira NG Blackstone EH. Postcardiotomy mechanical support: risk factors and outcomes. Ann Thorac Surg. (2001) 71(3 Suppl):S60–6. discussion S82–5. 10.1016/S0003-4975(00)02626-6 11265868

[3] Sklar MC Sy E Lequier L Fan E Kanji HD. Anticoagulation practices during venovenous extracorporeal membrane oxygenation for respiratory failure. A systematic review. Ann Am Thorac Soc. (2016) 13(12):2242–50. 10.1513/AnnalsATS.201605-364SR 27690525

[4] Yang XT Chen YJ Zeng H Deng L Chang L Li Y Successful treatment of large left ventricular thrombosis during extracorporeal membrane oxygenation (ECMO): a case report and review of the literature. Clin Case Rep. (2025) 13(1):e70123. 10.1002/ccr3.70123 39839947 PMC11748204

[5] Wang YD Lin JF Huang XY Han XD. Successful treatment of veno-arterial extracorporeal membrane oxygenation complicated with left ventricular thrombus by intravenous thrombolysis: a case report. World J Clin Cases. (2023) 11(14):3323–9. 10.12998/wjcc.v11.i14.3323 37274033 PMC10237126

[6] Yoshida Y Nakanishi K Jin Z Daimon M Ishiwata J Sawada N Association between progression of arterial stiffness and left ventricular remodeling in a community-based cohort. JACC Adv. (2023) 2(5):100409. 10.1016/j.jacadv.2023.100409 38938996 PMC11198086

[7] Saheera S Krishnamurthy P. Cardiovascular changes associated with hypertensive heart disease and aging. Cell Transplant. (2020) 29:963689720920830. 10.1177/0963689720920830 32393064 PMC7586256

[8] Nakamura E Aoki T Endo Y Kazmi J Hagiwara J Kuschner CE Organ-Specific mitochondrial alterations following ischemia-reperfusion injury in post-cardiac arrest syndrome: a comprehensive review. Life (Basel). (2024) 14(4):477. 10.3390/life14040477 38672748 PMC11050834

[9] Masiero G Cardaioli F Rodinò G Tarantini G. When to achieve complete revascularization in infarct-related cardiogenic shock. J Clin Med. (2022) 11(11):3116. 10.3390/jcm11113116 35683500 PMC9180947

[10] Sun M Zong Q Ye LF Fan Y Yang L Lin R. Prognostic factors in children with acute fulminant myocarditis receiving venoarterial extracorporeal membrane oxygenation. World J Pediatr Surg. (2022) 5(1):e000271. 10.1136/wjps-2021-000271 36474629 PMC9717374

[11] Cevasco M Takayama H Ando M Garan AR Naka Y Takeda K. Left ventricular distension and venting strategies for patients on venoarterial extracorporeal membrane oxygenation. J Thorac Dis. (2019) 11(4):1676–83. 10.21037/jtd.2019.03.29 31179113 PMC6531683

[12] Pavlushkov E Berman M Valchanov K. Cannulation techniques for extracorporeal life support. Ann Transl Med. (2017) 5(4):70. 10.21037/atm.2016.11.47 28275615 PMC5337209

[13] Pergola V Cameli M Mattesi G Mushtaq S D'Andrea A Guaricci AI Multimodality imaging in advanced heart failure for diagnosis, management and follow-up: a comprehensive review. J Clin Med. (2023) 12(24):7641. 10.3390/jcm12247641 38137711 PMC10743799

[14] Na SJ Yang JH Yang JH Sung K Choi JO Hahn JY Left heart decompression at venoarterial extracorporeal membrane oxygenation initiation in cardiogenic shock: prophylactic versus therapeutic strategy. J Thorac Dis. (2019) 11(9):3746–56. 10.21037/jtd.2019.09.35 31656647 PMC6790473

[15] Siraw BB Isha S Mehadi AY Tafesse YT. In-hospital outcomes of cardiogenic shock patients: a propensity score-matched nationwide comparative analysis between intra-aortic balloon pump and percutaneous ventricular assist devices. Int J Cardiol. (2025) 427:133093. 10.1016/j.ijcard.2025.133093 40044046

[16] Lorusso R Raffa GM Alenizy K Sluijpers N Makhoul M Brodie D Structured review of post-cardiotomy extracorporeal membrane oxygenation: part 1-adult patients. J Heart Lung Transplant. (2019) 38(11):1125–43. 10.1016/j.healun.2019.08.014 31522913 PMC8152367

[17] Ezad SM Ryan M Donker DW Pappalardo F Barrett N Camporota L Unloading the left ventricle in venoarterial ECMO: in whom, when, and how? Circulation. (2023) 147(16):1237–50. 10.1161/CIRCULATIONAHA.122.062371 37068133 PMC10217772

[18] Bernhardt AM Potapov E Vandenbriele C Skurk C Bertoldi LF Pappalardo F. Differential utilization of impella devices, extracorporeal membrane oxygenation, and combined therapies as escalation and de-escalation strategies. Eur Heart J Suppl. (2023) 25(Suppl I):I32–i8. 10.1093/eurheartjsupp/suad131 38093771 PMC10715938

[19] Abuelazm M Nawlo A Ibrahim AA Amin AM Mahmoud A Elshenawy S Early left ventricular unloading during extracorporeal membrane oxygenation in cardiogenic shock: a systematic review and meta-analysis. Artif Organs. (2025) 49(4):556–70. 10.1111/aor.14898 39494489 PMC11974487

[20] Attinger-Toller A Bossard M Cioffi GM Tersalvi G Madanchi M Bloch A Ventricular unloading using the impella(TM) device in cardiogenic shock. Front Cardiovasc Med. (2022) 9:856870. 10.3389/fcvm.2022.856870 35402561 PMC8984099

[21] Watanabe S Fish K Kovacic JC Bikou O Leonardson L Nomoto K Left ventricular unloading using an impella CP improves coronary flow and infarct zone perfusion in ischemic heart failure. J Am Heart Assoc. (2018) 7(6):e006462. 10.1161/JAHA.117.006462 29514806 PMC5907535

[22] De Lazzari B Capoccia M Badagliacca R Bozkurt S De Lazzari C. IABP Versus impella support in cardiogenic shock: “in silico” study. J Cardiovasc Dev Dis. (2023) 10(4):140. 10.3390/jcdd10040140 37103019 PMC10198323

[23] AlGhamdi M Saiydoun G Lebreton G Mazzucotelli JP. Percutaneous atrial septostomy for left ventricular unloading in patients on peripheral venoarterial extracorporeal membrane oxygenation: a systematic review and meta-analysis. Am Heart J Plus. (2025) 54:100542. 10.1016/j.ahjo.2025.100542 40276546 PMC12019464

[24] Hála P Kittnar O. Hemodynamic adaptation of heart failure to percutaneous venoarterial extracorporeal circulatory supports. Physiol Res. (2020) 69(5):739–57. 10.33549/physiolres.934332 32901493 PMC8549913

[25] Meani P Gelsomino S Natour E Johnson DM Rocca HB Pappalardo F Modalities and effects of left ventricle unloading on extracorporeal life support: a review of the current literature. Eur J Heart Fail. (2017) 19(Suppl 2):84–91. 10.1002/ejhf.850 28470925

[26] Sacks D Baxter B Campbell BCV Carpenter JS Cognard C Dippel D Multisociety consensus quality improvement revised consensus statement for endovascular therapy of acute ischemic stroke. Int J Stroke. (2018) 13(6):612–32. 10.1177/1747493018778713 29786478

[27] Streng AS Delnoij TSR Mulder MMG Sels J Wetzels RJH Verhezen PWM Monitoring of unfractionated heparin in severe COVID-19: an observational study of patients on CRRT and ECMO. TH open. (2020) 4(4):e365–e75. 10.1055/s-0040-1719083 33235946 PMC7676995

[28] Byun JH Jang IS Kim JW Koh EH. Establishing the heparin therapeutic range using aPTT and anti-Xa measurements for monitoring unfractionated heparin therapy. Blood Res. (2016) 51(3):171–4. 10.5045/br.2016.51.3.171 27722127 PMC5054248

[29] Nguyen TP Phan XT Huynh DQ Viet Truong HT Hai Le YN Nguyen TM Monitoring unfractionated heparin in adult patients undergoing extracorporeal membrane oxygenation (ECMO): aCT, APTT, or ANTI-XA? Crit Care Res Pract. (2021) 2021:5579936. 10.1155/2021/5579936 34055407 PMC8112950

[30] Li T Zhailauova A Kuanyshbek A Wachruschew I Tulegenov S Sazonov V Heparin resistance in patients receiving extracorporeal membrane oxygenation: a review. J Clin Med. (2024) 13(24):7633. 10.3390/jcm13247633 39768556 PMC11728406

[31] Lardinois B Hardy M Michaux I Horlait G Rotens T Jacqmin H Monitoring of unfractionated heparin therapy in the intensive care unit using a point-of-care aPTT: a comparative, longitudinal observational study with laboratory-based aPTT and anti-Xa activity measurement. J Clin Med. (2022) 11(5):1338. 10.3390/jcm11051338 35268436 PMC8911237

[32] Levy JH Frere C Koster A. Resistance to unfractionated heparin in the ICU: evaluation and management options. Intens Care Med. (2023) 49(8):1005–7. 10.1007/s00134-023-07103-x PMC10242222 37278759

[33] Mehmedagić A Skrbo S Softić D Haracić M. *In vitro* modeling of the influence of FVIII activity and heparin induced prolongation of APTT. Bosnian J Basic Med Sci. (2005) 5(3):26–9. 10.17305/bjbms.2005.3266 PMC7202147 16351577

[34] Shakhidzhanov S Filippova A Bovt E Gubkin A Sukhikh G Tsarenko S Severely ill COVID-19 patients may exhibit hypercoagulability despite escalated anticoagulation. J Clin Med. (2025) 14(6):1966. 10.3390/jcm14061966 40142778 PMC11943368

[35] Novelli C Borotto E Beverina I Punzi V Radrizzani D Brando B. Heparin dosage, level, and resistance in SARS-CoV2 infected patients in intensive care unit. Int J Lab Hematol. (2021) 43(6):1284–90. 10.1111/ijlh.13543 33855802 PMC8251410

[36] Sun L Zhao J Jiang G Lu W Rong H Lun L In-house chromogenic anti-factor Xa assay: development, validation, and identification of factors predicting APTT discordance. Front Med (Lausanne). (2025) 12:1745447. 10.3389/fmed.2025.1745447 41601727 PMC12832324

[37] Sacco A Morici N Oreglia JA Tavazzi G Villanova L Colombo C Left ventricular unloading in acute on chronic heart failure: from statements to clinical practice. J Pers Med. (2022) 12(9):1463. 10.3390/jpm12091463 36143247 PMC9502778

