# Supplementary material for: Case Report: Extracorporeal membrane oxygenation in acute coronary syndrome: a rare case of massive left ventricular thrombus
Source: Front Cardiovasc Med. 2026 Mar 16;13:1778609. doi: 10.3389/fcvm.2026.1778609 (PMC13034136; doi:10.3389/fcvm.2026.1778609)
Supplement: Supplementary file 1 [file Table1.docx]

| **No.** | **Parameter (English)** | **Result** | **Unit** |
| --- | --- | --- | --- |
| 1 | R (Coagulation Factor Activity) | 12.0 min | 5–10 min (shorter = higher activity) |
| 2 | K (Fibrinogen Function) | 2.8 min | 1–3 min (shorter = higher function) |
| 3 | Angle (Fibrinogen Function) | 46.1 deg | 55–78° (higher = higher function) |
| 4 | MA (Platelet Function) | 66.8 mm | 51–75 mm (higher = higher function) |
| 5 | CI (Coagulation Index) | –5.0 | — |
| 6 | EPL (Fibrinolysis Indicator) | 0.0 % | 0–15 % (>15 % = hyper-fibrinolysis) |
| 7 | LY30 (Fibrinolysis Indicator) | 0.0 % | 0–8 % (>8 % = hyper-fibrinolysis) |

**Supplemental Table 1** Thromboelastogram on the third postoperative day.
